# CHOICE IN DIRECTLY-FUNDED HOME CARE

**DOI:** 10.1093/geroni/igae098.2144

**Published:** 2024-12-31

**Authors:** Lisette Dansereau, Christine Kelly

**Affiliations:** University of Manitoba, Winnipeg, Manitoba, Canada; University of Manitoba, Winnipeg, Manitoba, Canada

## Abstract

Directly funded (DF) home care, also referred to as direct payments or cash-for care, gives clients (self-managers) or unpaid caregivers (family-managers) a budget to choose their own home support services. DF research tends to be dominated by the assumption that choice is inherently beneficial, and rarely attends to the circumstances or experiences of family-managers. Using feminist care theory and Mol’s ‘logic of care’ as an analytical lens, this study aims to address this gap through a nuanced approach to the concept of choice in care services. The findings are based on focus group discussions held between June-July 2021 with 32 participants using DF home care in Manitoba, Canada. Qualitative thematic analysis was conducted to answer the question “how do DF users experience the tensions of choice, risk and responsibility?” The first theme demonstrates that choice in DF results in high quality care. The second theme illuminates some of the risks involved in exercising choice, particularly when an individual’s responsibility for coordinating formal services is unsupported. The final theme shows that family-managers tend to take on the responsibility of DF not as a first choice but as a last resort after experiencing other care options as substandard and to avoid institutional care for an older adult. We conclude that family-management in DF places new formal responsibilities on informal caregivers, often without support. We recommend reducing private responsibility in the context of DF, and identify four domains where there is potential to enhance choice in professionally-organized home care services.

